# Metagenomic Identification of *Fusarium solani* Strain as Cause of US Fungal Meningitis Outbreak Associated with Surgical Procedures in Mexico, 2023

**DOI:** 10.3201/eid3105.241657

**Published:** 2025-05

**Authors:** Charles Y. Chiu, Venice Servellita, Mikael de Lorenzi-Tognon, Patrick Benoit, Nanami Sumimoto, Abiodun Foresythe, Filipe M. Cerqueira, Natalie Williams-Bouyer, Ping Ren, Lauren Nicholas S. Herrera, David C. Gaston, Leanna Sayyad, Shannon L. Whitmer, John Klena, Holenarasipur R. Vikram, Jeremy A.W. Gold, Lalitha Gade, Lindsay Parnell, Elizabeth Misas, Tom M. Chiller, Isabel S. Griffin, Sridhar V. Basavaraju, Dallas J. Smith, Anastasia P. Litvintseva, Nancy A. Chow

**Affiliations:** Chan-Zuckerberg Biohub, San Francisco, California, USA (C.Y. Chiu); University of California San Francisco, San Francisco (C.Y. Chiu, V. Servellita, M. de Lorenzi-Tognon, P. Benoit, N. Sumimoto, A. Foresythe); Abbott Pandemic Defense Coalition, Abbott Park, Illinois, USA (C.Y. Chiu, V. Servellita, M. de Lorenzi-Tognon, P. Benoit, N. Sumimoto, A. Foresythe); The University of Texas Medical Branch at Galveston, Galveston, Texas, USA (F.M. Cerqueira, N. Williams-Bouyer, P. Ren); Vanderbilt University Medical Center, Nashville, Tennessee, USA (L.N.S. Herrera, D.C. Gaston); Centers for Disease Control and Prevention, Atlanta, Georgia, USA (L. Sayyad, S.L. Whitmer, J. Klena, J.A.W. Gold, L. Gade, L. Parnell, E. Misas, T.M. Chiller, I.S. Griffin, S.V. Basavaraju, D.J. Smith, A.P. Litvintseva, N.A. Chow); Mayo Clinic, Phoenix, Arizona, USA (H.R. Vikram)

**Keywords:** *Fusarium solani*, fungi, meningitis/encephalitis, metaMELT, multiple extended locus typing, metagenomic next-generation sequencing, mNGS, agnostic pathogen detection, outbreak investigation, *Nectria haematococca*, SURPI+ computational pipeline for pathogen detection, United States, Mexico

## Abstract

We used metagenomic next-generation sequencing (mNGS) to investigate an outbreak of *Fusarium solani* meningitis in US patients who had surgical procedures under spinal anesthesia in Matamoros, Mexico, during 2023. Using a novel method called metaMELT (metagenomic multiple extended locus typing), we performed phylogenetic analysis of concatenated mNGS reads from 4 patients (P1–P4) in parallel with reads from 28 fungal reference genomes. Fungal strains from the 4 patients were most closely related to each other and to 2 cultured isolates from P1 and an additional case (P5), suggesting that all cases arose from a point source exposure. Our findings support epidemiologic data implicating a contaminated drug or device used for epidural anesthesia as the likely cause of the outbreak. In addition, our findings show that the benefits of mNGS extend beyond diagnosis of infections to public health outbreak investigation.

Whole-genome sequencing (WGS) of pathogens, including viruses, bacteria, and fungi, is a vital tool for detecting and investigating outbreaks (*1*–*3*). In addition to providing definitive identification of the causative organism, WGS can be used to construct phylogenetic trees, which in turn can be used to detect more cases, resolve disease transmission patterns, and identify potential outbreak sources. However, conventional WGS depends on the ability to grow the pathogen in culture. For fastidious organisms such as mycobacteria, fungi, and some viruses, successful culturing is often difficult and slow; growth of the organism requires weeks to months and sometimes is not possible at all. To bypass the requirement for culture, techniques such as multilocus sequence typing/multilocus sequence analysis (MLST/MLSA) have been developed, whereby specific regions of the microbial genome (loci) are targeted for PCR amplification directly from primary clinical material (*4*,*5*). A key limitation of MLST/MLSA, however, is the need to have the target organism identified, loci defined, and primers at hand a priori. Thus, MLST/MLSA is typically not useful for immediate outbreak situations caused by rare or unexpected pathogens.

Metagenomic next-generation sequencing (mNGS) is an agnostic diagnostic method with the potential to identify any pathogen, whether bacterial, viral, parasitic, or fungal, on the basis of shotgun sequencing of DNA or RNA (*6*). The use of mNGS testing from clinical samples has been shown to increase diagnostic yield and provide actionable results in multiple prospective studies (*7*–*11*). In addition, mNGS has proven to be an invaluable tool for identifying and initially characterizing emerging pathogens, such as SARS-CoV-2 (*12*). However, the use of mNGS to date has been mainly focused on pathogen detection for clinical diagnostic purposes, rather than outbreak investigation, because coverage of the pathogen genome recovered by mNGS is generally sparse and uneven.

On May 8, 2023, the Centers for Disease Control and Prevention (CDC) Emerging Infections Network reported cases of suspected fungal meningitis in US patients who had undergone surgical procedures performed under epidural anesthesia in the city of Matamoros in Durango state, Mexico (*13*). Using clinical mNGS testing, the University of California, San Francisco (UCSF), clinical microbiology laboratory reported identification of *Fusarium solani* species complex in cerebrospinal fluid from an affected patient (P1) with probable fungal meningitis in the United States on May 28, 2023 (*14*). That initial case and additional cases were subsequently confirmed independently by panfungal PCR followed by sequencing of the amplicon to confirm detection of *F. solani* (*13*). During the outbreak, a total of 184 patients in 22 US states were identified as persons potentially exposed, among whom 24 were identified with fungal meningitis and 12 died, mainly from severe vascular complications (*13*,*15*). Here, we describe a novel analytic technique called metaMELT, or metagenomic multiple extended locus typing, as a tool for simultaneously diagnosing infections and characterizing the interrelatedness of *F. solani* strains in patients from the Matamoros outbreak.

## Methods

### Human Sample Collection and Processing

Cerebrospinal fluid (CSF) samples from 5 patients (P1–P5) in the Matamoros outbreak were available for analysis. A CSF sample from patient P1 was sent to the UCSF Clinical Microbiology Laboratory for CSF mNGS testing and was processed and sequenced as previously described (*16*). Residual CSF, plasma, and brain tissue biopsy samples from patients P1­–P4 were also processed and sequenced using mNGS. Cultures of *F. solani* were also obtained from P1 and P5 CSF samples. 

For P1­–P4 samples, we extracted DNA by using the MagMAX Viral/Pathogen II (MVP II) Nucleic Acid Isolation Kit (Thermo Fisher Scientific, https://www.thermofisher.com) and the KingFisher Flex Purification System with a 96 deep-well head (Thermo Fisher Scientific). We loaded the extracted DNA on the MagicPrep NGS instrument (Tecan Genomics, Inc., https://www.tecan.com) to undergo end repair, adaptor ligation and barcoding, amplification (25 cycles), and purification. We enriched some of the libraries for microbial DNA by using DepleteX (Jumpcode Genomics, https://www.jumpcodegenomics.com), an early release of a CRISPR-based human DNA depletion kit, according to the manufacturer’s specifications. That kit leverages Cas9 depletion and exonuclease activity to efficiently remove human DNA from samples with high human content. 

CRISPR-based host depletion increased the number of *Fusarium*-specific reads in the P1 CSF DNA library from 13 to 223 (16.4-fold enrichment increase), corresponding to an increase of 1.5 to 2.4 reads per million (RPM), a 1.6-fold enrichment increase. In the P3 plasma DNA library, the number of reads decreased from 60 to 36 after enrichment, but that decrease corresponded to an RPM increase from 0.12 to 0.16 (1.3-fold enrichment increase). We quantified and normalized the libraries by using the Qubit dsDNA HS Assay on the Qubit Flex (Thermo Fisher Scientific). We sequenced final pooled libraries as single-end reads on the NextSeq 550 (Illumina, https://www.illumina.com) by using the High-Output Kit or the NovaSeq Kit (Illumina) at 150 cycles.

### *F. solani* WGS

We performed WGS of 18 *F. solani* isolates, including isolates cultured from 2 patients (P1 and P5) in the Matamoros outbreak as follows. We extracted DNA by using the DNeasy Blood and Tissue kit (QIAGEN, https://www.qiagen.com), and then used the NEBNext Ultra DNA Library Prep kit (New England Biolabs, https://www.neb.com) to construct DNA fungal genomic libraries for Illumina sequencing. We sequenced the isolate from patient P1 (genome B27264) on the Illumina MiSeq (250-bp paired-end sequencing, or 500 cycles). We sequenced the isolate from patient P5 (genome B27166) and 16 additional *F. solani* genomes unrelated to the Matamoros outbreak by using the NovaSeq 6000SP Reagent Kit (Illumina) at 500 cycles.

### Bioinformatic Methods

We used the SURPI+ computational pipeline version 1.0.0 (https://github.com/chiulab/SURPI-plus-dist), run as a container on either a secure server or cloud infrastructure, to identify pathogens from mNGS data (*16*,*17*). We preprocessed reads by trimming adapters and removing low-complexity and low-quality sequences and then performed computational subtraction of human reads by using SNAP version 1.0 (*16*) and Bowtie2 version 2.3.2 GTCh38/hg38 build (https://sourceforge.net/projects/bowtie-bio) to align and exclude reads mapping to the human genome. We aligned the remaining nonhuman reads to microbial reference sequences in the National Center for Biotechnology Information (NCBI) nucleotide database (https://www.ncbi.nlm.nih.gov/nucleotide; March 2019 build) using the SNAP nucleotide aligner (*18*). We performed filtering and taxonomic classification algorithms to remove spurious hits and classify each read at the species, genus, or family level, as previously described (*16*). 

We further analyzed CSF mNGS reads from P1 by nucleotide BLASTn (https://blast.ncbi.nlm.nih.gov) alignment to the genome of an *F. solani* reference strain (ATCC strain no. MYA-4622) with an E-value of 1 × 10^−8^ and the remaining parameters set to corresponding default values (*19*). We visualized mapped reads by using Ensembl Fungi (*20*). Among the 28 *F. solani* cultured isolates included in the analysis, 10 were from genomes downloaded from GenBank. For the other 18 newly sequenced isolates, we obtained draft genomes by de novo assembly of raw reads using the SPADES genome assembler version 3.15.4 at default parameters (*21*).

We wrote Linux scripts and code to perform the metaMELT method for each patient as follows. For reads from patients P1–P4 identified as *F. solani* in SURPI+, we trimmed to the desired length and successively aligned reads by using BLASTn at a 1 × 10^−8^ E-value cutoff for each of the 28 *F. solani* reference genomes, including the assembled genomes from P1 and P5. If an alignment was successful, we extracted the mapped region corresponding to the read from the reference genome; otherwise, the system generated a synthetic dummy read consisting of a stretch of Ns (ambiguous nucleotides). We then concatenated reads from all 4 patients and the corresponding mapped regions in each reference genome by using a 50-bp spacer of Ns to separate each read or region. We performed multiple sequence alignment of the concatenated sequences by using MAFFT version 7.388 (*22*) and the following parameters: FFT-NS-2 algorithm, 200 PAM/k = 2 scoring matrix, gap open penalty = 3, and offset value = 0.123. We then constructed phylogenetic trees by using PhyML 3.0 (*23*) and substitution model TN93 with SH-like branch supports, in Geneious version 11.1.5 (*24*).

### Inclusion and Ethics

Sequencing data from clinical CSF mNGS testing and deidentified residual samples from hospitalized patients who were part of the *F. solani* outbreak were analyzed under protocols approved by the UCSF institutional review board (protocol no. 11-05519). Clinical and demographic patient-level data were not collected because that information was not considered relevant to this study. All patients with sufficient remaining volume of residual samples were included in the study.

## Results

**Figure 1:**
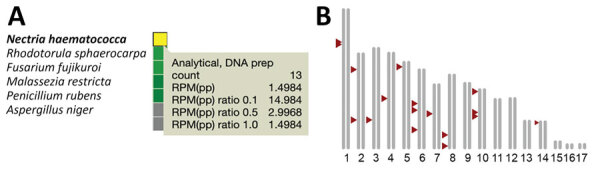
Identification of *Fusarium solani* in CSF from a patient with fungal meningitis associated with surgical procedures in Matamoros, Mexico, 2023. A) Heat map of 13 mNGS CSF reads from patient P1 showing reads aligning to *Nectria hematococca,* the anamorph of *F. solani.* That initial detection triggered a Centers for Disease Control and Prevention notification recommending testing for all patients in the outbreak who might have been exposed (*14*). B) Mapping of the 18 total *Fusarium* spp. mNGS reads recovered from patient P1 to the *F. solani* reference genome (ATCC MYA-4622). Arrows indicate the mapped positions of the 18 reads. CSF, cerebrospinal fluid; mNGS, metagenomic next-generation sequencing; RPM(pp), reads per million (preprocessed).

We used clinical CSF mNGS testing and SURPI+ analysis to diagnose *F. solani* infection in case P1. Among the 8.7 million reads that we recovered from CSF, 13 (0.00015%) aligned most closely to the genome of *Nectria hematococca*, the anamorph of *F. solani* (Figure 1, panel A). We did not observe any co-infections from viruses, bacteria, or parasites. The default SURPI+ pipeline uses the GenBank nucleotide database for its microbial reference database (*16*,*17*), which does not contain any fungal whole-genome sequences. Because fungal whole-genome sequences are found in the GenBank whole-genome shotgun database (*25*), we used that database to align the mNGS reads directly to an *F. solani* reference genome (ATCC strain no. MYA-4622) and identified an additional 5 fungal reads. We found that the 18 total mapped *F. solani* reads were distributed randomly across the ≈53 Mb genome (Figure 1, panel B). 

We subsequently obtained more *F. solani* reads from the P1 CSF sample by additional sequencing and we also sequenced CSF, plasma, or brain tissue from 3 more patients (P2, P3, and P4) in the Matamoros outbreak who had PCR-confirmed *F. solani* meningitis. To maximize recovery of *F. solani* reads, we sequenced mNGS libraries both without and with enrichment for microbial DNA using DepleteX, although the level of enrichment was only 1.3–1.6 RPM using that kit. The total number of *F. solani* reads recovered from each patient ranged from 263 to 187,773.

**Figure 2:**
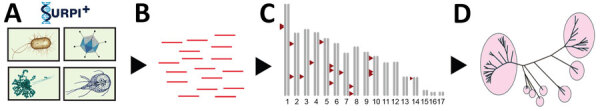
Flowchart showing the metaMELT analysis workflow used for identification of *Fusarium solani* strain as cause of fungal meningitis US outbreak associated with surgical procedures in Mexico, 2023. metaMELT (metagenomic multiple extended locus typing), is a novel analytic technique for simultaneously diagnosing the infection and characterizing the interrelatedness of *F. solani* strains. metaMELT uses the following steps: A) perform mNGS analysis of patient samples (i.e., cerebrospinal fluid, plasma, or brain tissue), using the SURPI+ computational pipeline (https://github.com/chiulab/SURPI-plus-dist) to identify pathogens; B) identify *F. solani* reads; C) map reads to the *F. solani* reference genome and then extract and concatenate; D) perform phylogenetic analysis on concatenated sequences. SURPI, sequence-based ultra-rapid pathogen identification.

**Figure 3:**
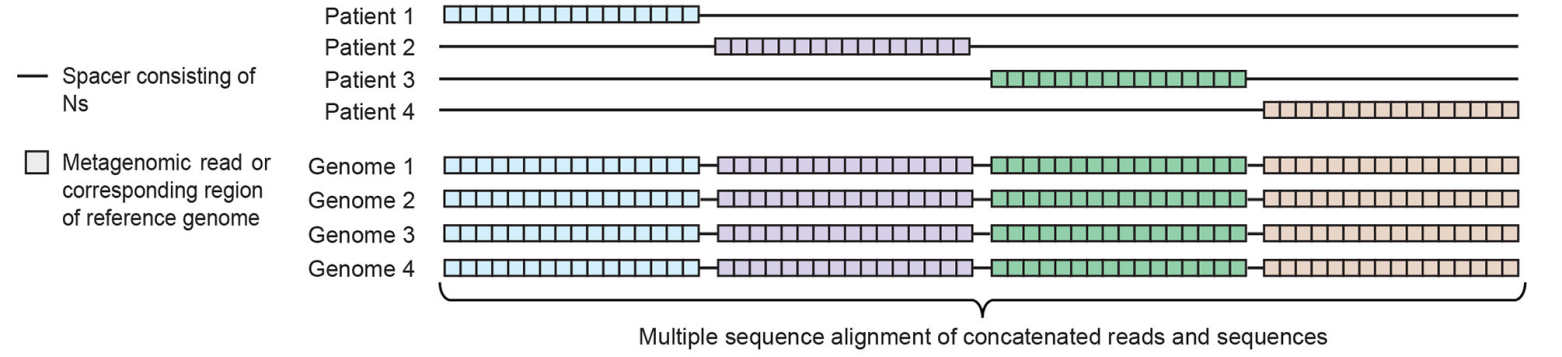
Diagram of concatenation and alignment step of the metaMELT procedure (metagenomic multiple extended locus typing, a novel analytic technique for simultaneously diagnosing the infection and characterizing the interrelatedness of *Fusarium solani* strains) used for identification of *F. solani* strain as cause of fungal meningitis US outbreak associated with surgical procedures in Mexico, 2023. The diagram shows concatenated metagenomic next-generation sequencing reads from 4 patients and the corresponding regions extracted from reference genomes, which are aligned to by using MAFFT version 7.388 (*22*). This diagram demonstrates the steps shown in Figure 2, panels C–D.

The number of fungal mNGS reads was not sufficient to assemble the *F. solani* genome from any single patient. To achieve 30× coverage, we estimated that ≈10.6 million 150-bp *F. solani*–specific reads would need to be sequenced. The actual read numbers and percent coverage achieved from CSF mNGS from infected patients was extremely low; the number of reads ranged from 263 to 187,773 and the coverage ranged from 0.0005% to 0.35% of the fungal genome (Table 1). Thus, we developed metaMELT as a tool to enable comparison of fungal strains from individual patients using sparse mNGS data. The metaMELT method involves first extracting the regions defined by randomly selected mNGS reads from each patient sample from their corresponding locations (loci) in all available *F. solani* reference genomes (n = 28) (Figure 2). The total number of extracted reads or loci is equal to the product of the number of samples and the number of mNGS reads that are randomly selected from each sample. After concatenating the extracted reads, we performed phylogenetic analysis of the concatenated sequences, with each sequence derived from a single patient or a reference genome (Figure 3).

**Table 1:** Number of reads recovered from CSF, plasma, or brain tissue from 4 patients used for identification of *Fusarium solani* strain as cause of fungal meningitis US outbreak associated with surgical procedures in Mexico, 2023*

Patient ID	Location, USA	Sample type (no. reads)
P1†‡	State 1	CSF (263)
P2‡	State 2	CSF (187,773)
P3‡	State 2	CSF (2,669), plasma (96)
P4	State 2	CSF (4), brain tissue (608)

*Reads were recovered by using mNGS. CSF, cerebrospinal fluid; ID, identification; mNGS, metagenomic next-generation sequencing. †Newly diagnosed case by mNGS testing of CSF. ‡Fatal case.

**Figure 4:**
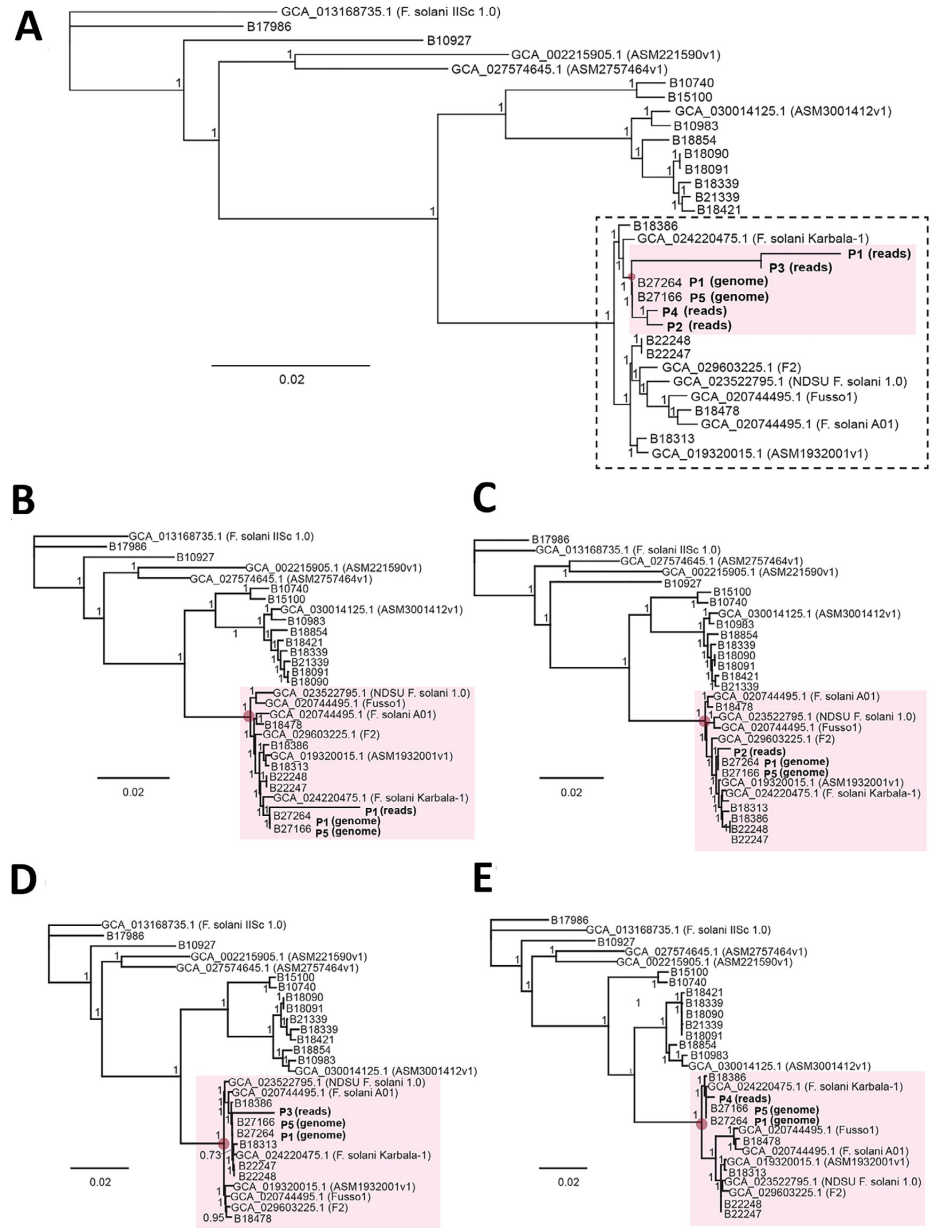
Phylogenetic analysis of concatenated metagenomic next-generation sequencing reads from US patients from a fungal meningitis outbreak associated with surgical procedures in Mexico, 2023. A) Phylogenetic trees showing clustering of strains from patients P1–P5 (pink shaded region) within a subclade that also includes fungal genomes unrelated to the outbreak (dotted rectangle). B–E) Phylogenetic trees of individual patients exhibiting similar topologies: B) P1; C) P2; D) P3; E) P4. Each patient is positioned in a cluster containing the same reference genomes, including the 2 outbreak genomes recovered from patients P1 and P5. Outbreak reads were mapped to corresponding regions from *Fusarium solani* reference genomes by using metaMELT (metagenomic multiple extended locus typing, a novel analytic technique for simultaneously diagnosing the infection and characterizing the interrelatedness of *F. solani* strains). Scale bars indicate nucleotide substitutions per site. P1–P5, patients 1­–5.

By early 2024, only 10 complete *F. solani* reference genomes were available for download in the GenBank whole-genome shotgun database (Figure 4, panel A). We performed de novo assembly on additional draft genomes from 18 cultured *F. solani* isolates in the CDC fungal biorepository from raw next-generation sequence data and included those in the phylogenetic analysis (Figure 4, panel B). Each draft genome consisted of a series of contigs (*26*), ranging from 1,057 to 7,216 contigs (Table 2). Of note, 2 draft genomes, B27264 from P1 and B27166 from P5, had been newly assembled from cultured outbreak isolates; patient P5 was part of the Matamoros outbreak for whom residual CSF was not available for mNGS. The accuracy of phylogenetic analysis relied on polymorphisms between an outbreak strain or cultured isolate and each reference genome in the database and not on polymorphisms between individual strains or isolates, because the sparse mNGS reads recovered from the ≈53 Mb genome were unlikely to overlap.

**Table 2:** Data from de novo assembly of 18 draft genomes used for identification of *Fusarium solani* strain as cause of fungal meningitis US outbreak associated with surgical procedures in Mexico, 2023*

Genome assembly	No. contigs	Total contig length, bp	N50, bp
B10740	2,589	50,517,664	489,689
B10927	3,421	49,206,550	371,903
B10983	3,198	57,260,205	506,490
B15100	2,250	49,683,915	842,580
B17986	3,037	47,958,675	381,199
B18090	5,155	60,209,829	470,869
B18091	6,769	59,741,266	458,563
B18313	1,448	51,560,784	467,209
B18339	2,419	55,485,754	565,856
B18386	1,781	52,521,438	575,687
B18421	7,187	59,392,883	357,605
B18478	7,244	56,253,777	441,893
B18854	2,695	54,697,765	562,797
B21339	2,532	55,305,429	898,025
B22247	2,284	56,659,172	747,616
B22248	1,071	51,606,190	538,696
B27166	2,186	53,744,553	512,232
B27264	2,262	54,164,081	802,132

*bp, base pairs; N50, shortest contig length that needs to be included to cover 50% of the genome.

We performed phylogenetic analysis of the concatenated sequences from P1−P4 and the 28 reference genomes. To evaluate the flexibility of metaMELT for different sample types, P1 and P2 included CSF reads, P3 included only plasma reads (n = 96), and P4 included mostly brain biopsy tissue reads (608 of 612 reads). Each concatenated sequence consisted of 90 randomly selected mNGS reads of 150-bp length because only 96 total plasma mNGS reads were available for patient P3. The topology of the resulting tree revealed that the *F. solani* strains from all 5 patients in the Matamoros outbreak were positioned together in a single cluster (Figure 4, panel A). To account for potential bias in the phylogenetic estimates because of the high proportion of ambiguous nucleotides in each of the concatenated sequences from patients P1–P4 (*27*), we reran the phylogenetic analysis including only 1 patient at a time, and all trees positioned the individual patient in the same cluster (Figure 4, panels B–E).

**Figure 5:**
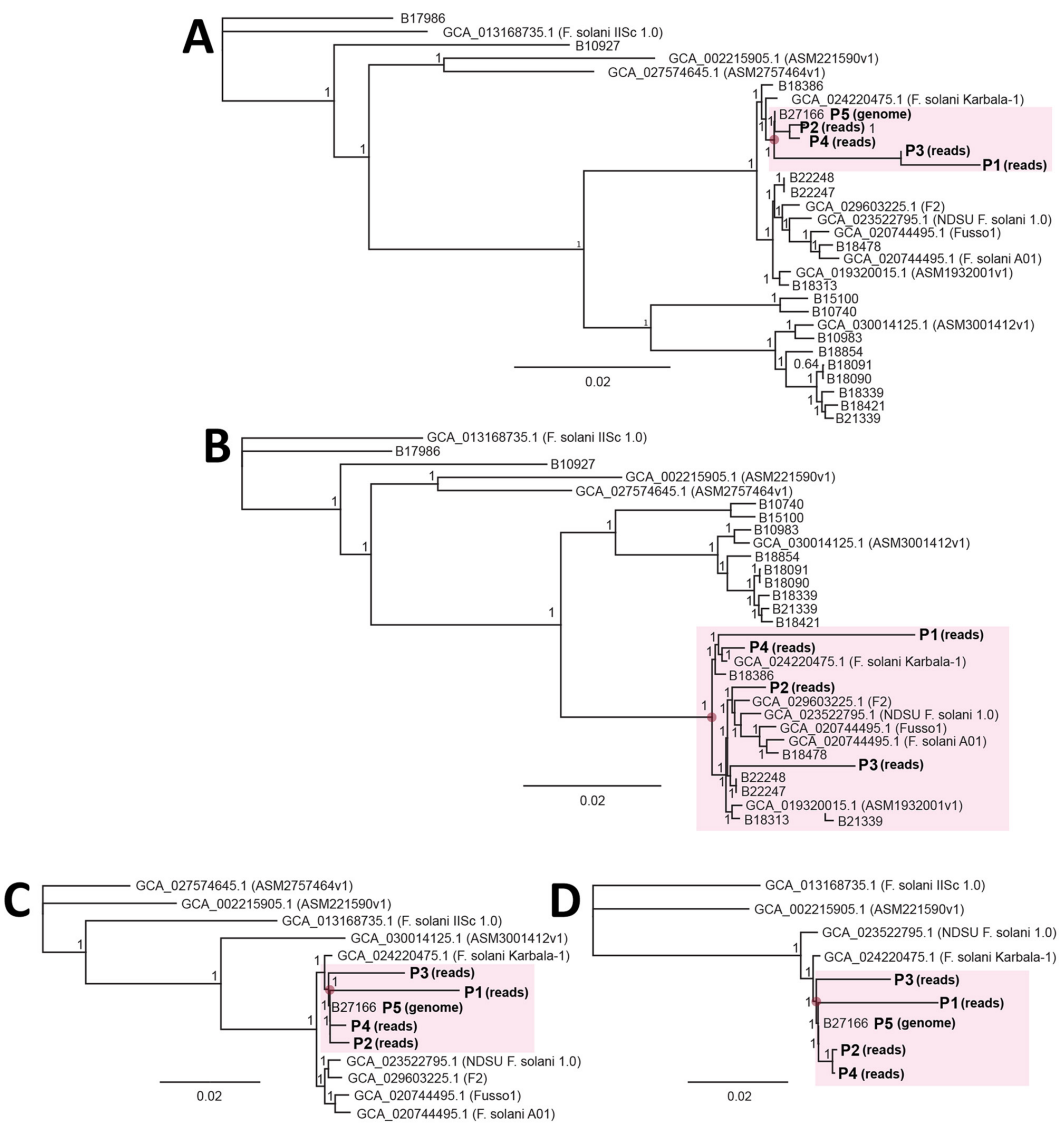
Effect of number of reference genomes on performance of metaMELT (metagenomic multiple extended locus typing, a novel analytic technique for simultaneously diagnosing the infection and characterizing the interrelatedness of *Fusarium solani* strains) for identification of *F. solani* strain as cause of fungal meningitis US outbreak associated with surgical procedures in Mexico, 2023. A, B) metaMELT phylogenetic trees that include mNGS reads from patients P1–P4 are shown with and without outbreak-related genomes: A) only 1 outbreak-related reference genome from P5; B) tree without any outbreak-related reference genomes. Note the clustering of patients P1–P4 (pink shaded regions) even in the absence of an outbreak-related reference genome. C, D) metaMELT phylogenetic trees that include mNGS reads from patients P1–P4 are shown with only 10 reference genomes, including patient P5 (C); and only 5 reference genomes, including patient P5 (D). Scale bars indicate nucleotide substitutions per site. P1–P5, patients 1­–5.

Next, we assessed whether clustering of mNGS reads from patients P1–P4 could still be visualized if only 1 outbreak-related reference genome from P5 was available for comparison or if no outbreak-related reference genome was available. We observed clustering of patients P1–P4 for both analyses (Figure 5, panels A, B), albeit with 11 unrelated genomes assigned to the cluster if no outbreak-related genome was included (Figure 5, panel B). Because only a few reference genomes might be in the existing reference databases for a given target pathogen, we then determined how the total number of available reference genomes affected patient clustering by metaMELT. Despite inclusion of only 10 or 5 total reference genomes, we still observed clustering of patients P1–P5 by phylogenetic analysis (Figure 5, panels C, D).

Based on MLST/MLSA analysis of the concatenated *ITS*, *rpb2*, and *tef1* genes (*28*), the predicted nucleotide pairwise identity between the 2 draft genomes from the Matamoros outbreak, B27264 (P1) and B27166 (P5), was 96% (Appendix Figure 1, panel A), and overall pairwise identities for the 28 reference genomes ranged from 89% to 96%. The high pairwise identity between the P1 and P5 isolates and positioning in the same subcluster by phylogenetic analysis (Appendix Figure 1, panel B) supported the notion of a single *F. solani* strain as the cause of the outbreak. Of note, the larger cluster consisting of an additional 11 genomes unrelated to the outbreak (Appendix Figure 1) was the same cluster that included the concatenated mNGS sequences from cases P1–P4 in all previous metaMELT phylogenetic analyses (Figures 4, 5).

We sought to ascertain the effect of read lengths and numbers on the clustering of the *F. solani* strains from patients P1−P5 by metaMELT. All 4 sequenced strains were correctly clustered at read lengths of >100 bp and >40 mNGS reads (Appendix Figure 2, panel A). To establish a quality control criterion for metaMELT at the empirically determined 40-read cutoff (Appendix Figure 2, panel A), we performed 20 bootstrap replicates in which we randomly sampled and analyzed 40 100-bp reads from patients P1–P4 by metaMELT. Phylogenetic trees from all 20 replicates correctly positioned patients P1–P4 in the same cluster (Appendix Figure 2, panel B).

## Discussion

In this study, we used clinical mNGS testing to identify *F. solani* in a patient from a fungal meningitis outbreak associated with surgical procedures in Matamoros, Mexico. We used a novel analytic method, metaMELT, to leverage the mNGS data to aid the public health investigation. The metaMELT method is a powerful tool for species identification and evaluation of the interrelatedness of outbreak strains for pathogens that are difficult to culture. Clinical samples from 4 infected patients (P1–P4) and cultured isolates from P1 and another patient from the outbreak (P5) clustered together by phylogenetic analysis, showing that the strains from all 5 patients are the same or closely related. Those results indicate a high likelihood that all 5 infections, and, by extension, the US outbreak, originated from a single point source. The results of the CDC epidemiologic investigation, taken together with our findings, suggest a single point source and implicate a contaminated drug or device used for epidural anesthesia, either at the site of manufacturing or from breakdown of infection control practices at the clinics, as the likely cause of the outbreak.

The minimum number of reads necessary for accurate metaMELT analyses is dependent on multiple factors, including the sequence diversity of the species’ genome, availability of closely related reference genomes, and the quality and length of reads. Given the high levels of sequence diversity and large number of potential outbreak pathogens, we propose an empirically derived quality control criterion for investigation of interrelatedness of outbreak samples that uses bootstrap sampling of randomly selected mNGS reads and a >95% accuracy cutoff (i.e., at least 19 of 20 trees with the correct topology) (Appendix Figure 2, panel B). 

The usefulness of metaMELT for analysis of the Matamoros outbreak described is likely due in part to the high sequence diversity of circulating *Fusarium* species isolates. In a previously published international outbreak of *F.* *keratitis* associated with contact lens wear, 19 of 39 isolates tested had a unique multilocus genotype (*29*). For other pathogens with lower genetic diversity, the usefulness of metaMELT may be limited because determining the relatedness of outbreak strains can be challenging. Another potential limitation of metaMELT is the need for enough complete reference genomes in publicly available databases for comparison, which might not be the case for rare or unusual pathogens.

Our study also extends the utility of mNGS and subsequent metaMELT analyses to not only agnostic diagnosis of infections in clinical settings but also the monitoring and tracking of communicable diseases, which are relevant to infection control, public health surveillance, and outbreak investigation. To date, those activities have largely relied on microbial WGS and phylogenetic analysis of the assembled sequences. However, WGS can be problematic because many atypical bacteria, fungi, and viruses grow slowly in culture or do not grow at all. The requirement for culture also inevitably delays the generation of actionable results in time-critical scenarios, such as outbreak investigation. Targeted methods, such as tiled multiplex amplicon sequencing for viruses (*30*) and MLST/MLSA for bacteria, parasites, and fungi (*4*,*5*,*31*,*32*), including *F. solani* (*28*,*33*), can be applied in lieu of WGS-based phylogenetic analysis. However, those approaches require that the pathogen be identified a priori and depend on the assays being immediately available at the time of identification, which is usually not the case with rare or unexpected pathogens such as *F. solani*. Clustering and phylogeny inferred from MLST/MLSA are also known to be inferior for some organisms compared to more detailed WGS analyses (*34*,*35*). Unlike WGS, metaMELT can be useful with mNGS reads that can vary by several orders of magnitude in number from sample to sample but typically produce very sparse coverage of the genome. In addition, metaMELT analysis enables the leveraging of mNGS data generated at the time of diagnosis and thus has the potential of providing immediate and actionable information to guide infection control and public health efforts.

In summary, our mNGS findings support epidemiologic data implicating a contaminated drug or device used for epidural anesthesia as a common point source and the likely cause of a *F. solani* meningitis outbreak in US patients associated with surgical procedures in Matamoros, Mexico. In addition, our findings show that mNGS could have benefits that extend beyond diagnosis of infections to more broadly assist in outbreak investigations.

Appendix Additional information on identification of *Fusarium solani* strain as cause of fungal meningitis US outbreak associated with surgical procedures in Mexico, 2023.
