# Supplementary material for: Metagenomic Identification of Fusarium solani Strain as Cause of US Fungal Meningitis Outbreak Associated with Surgical Procedures in Mexico, 2023
Source: Emerg Infect Dis. 2025 May;31(5):948–57. doi: 10.3201/eid3105.241657 (PMC12044249; doi:10.3201/eid3105.241657)
Supplement: Appendix — Additional information on identification of Fusarium solani strain as cause of fungal meningitis US outbreak associated with surgical procedures in Mexico, 2023. [file 24-1657-Techapp-s1.pdf]

Article DOI: https://doi.org/10.3201/eid3105.241657

EID cannot ensure accessibility for supplementary materials supplied by authors. Readers who have difficulty accessing supplementary content should contact the authors for assistance.

Metagenomic Identification of *Fusarium solani* Strain as Cause of US Fungal Meningitis Outbreak Associated with Surgical Procedures in Mexico, 2023

Appendix

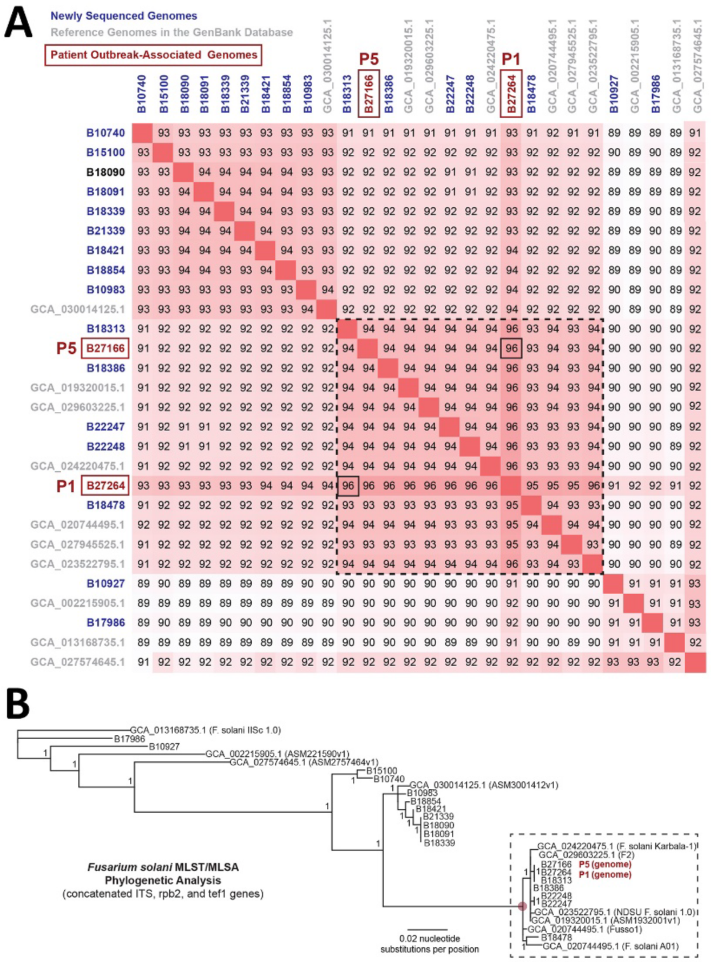

Appendix Figure 1. Pairwise distance matrix of average nucleotide identity used for identification of *Fusarium solani* strain as cause of fungal meningitis US outbreak associated with surgical procedures in Mexico, 2023. A) Pairwise distance matrix; B) phylogenetic tree based on the concatenated *ITS*, *rpb2*,

and *tef1* genes showing the positioning of the P1 and P5 genomes from the Matamoros outbreak in the same subcluster. We assessed pairwise distance of *F. solani* genomes based on MLST/MLSA analysis of the concatenated *ITS*, *rpb2*, and *tef1* genes. Concatenation of multiple genes was needed because *ITS* alone is inadequate for differentiation and classification of fungal species and strains (*1*). Note that the 2 fungal genomes, B27166 and B27264, recovered from patients P1 and P5, respectively, in the Matamoros outbreak share 96% pairwise identity in the matrix (A; black box). Other genomes unrelated to the outbreak also share 96% pairwise identity to B27166 and B27264 (dotted box) and are positioned together with the *F. solani* strains from patients P1-P5 in the Matamoros outbreak in the same subclade of the phylogenetic trees in Figure 4, panel A and Figure 5, panel B (dotted rectangles).

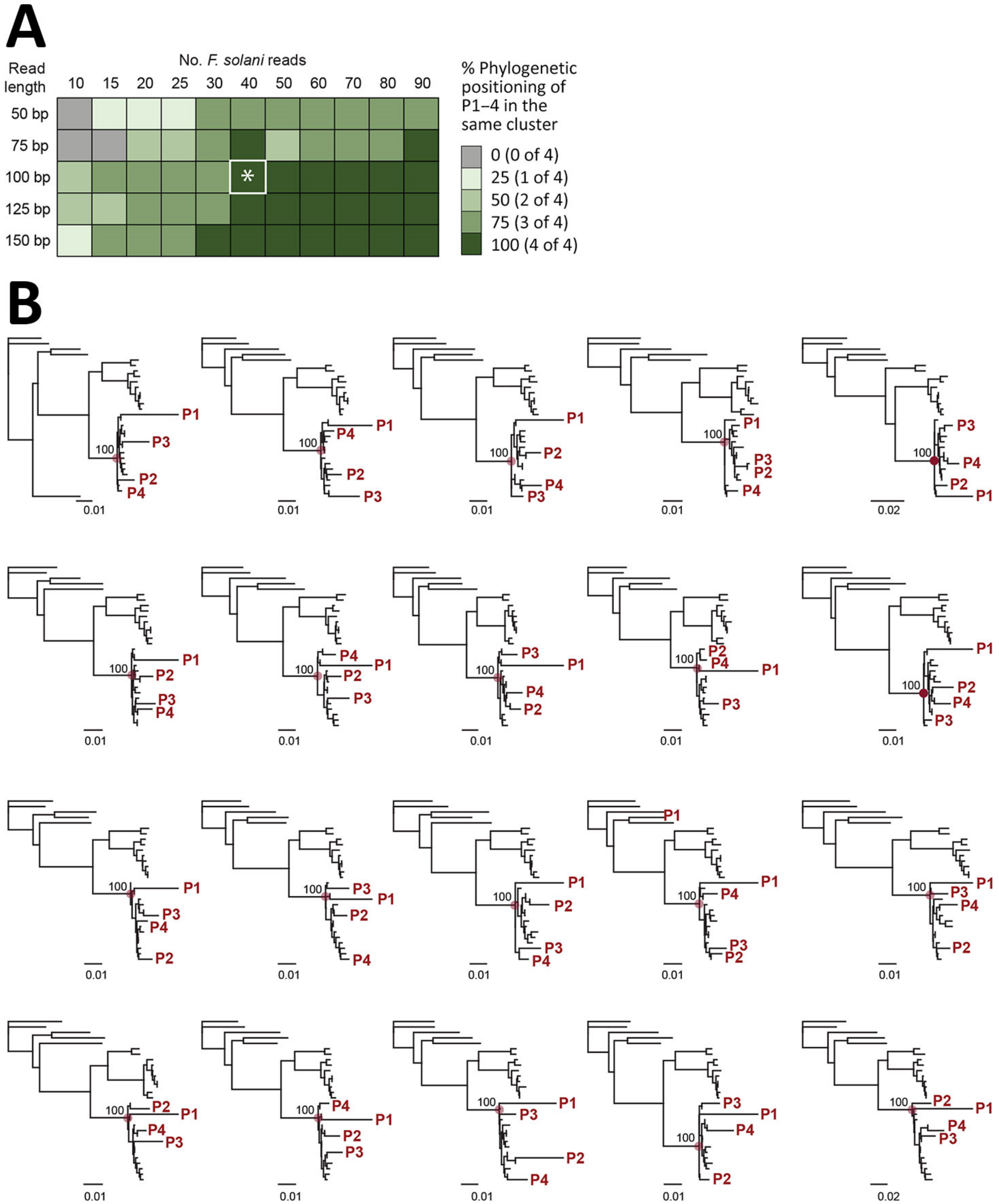

Appendix Figure 2. Effect of read numbers and lengths on performance of metaMELT (metagenomic multiple extended locus typing, a novel analytic technique for simultaneously diagnosing the infection and characterizing the interrelatedness of *Fusarium solani* strains) for identification of *F. solani* strain as cause of fungal meningitis US outbreak associated with surgical procedures in Mexico, 2023. A) Grid showing relationship between the number of reads and read lengths required for correct clustering of patients P1–

P4; asterisk (*) indicates reads used for the phylogenetic analysis shown in (B). B) Phylogenetic analysis of 40 randomly sampled 100-bp mNGS reads each from patients P1–P4, performed across 20 bootstrap replicates. In all 20 replicate trees, clustering of patients P1–P4 is observed. Scale bars indicate nucleotide substitutions per site. metaMELT, metagenomic multiple extended locus typing (a novel analytic technique for simultaneously diagnosing the infection and characterizing the interrelatedness of *F. solani* strains); mNGS, metagenomic next-generation sequencing; P1–P5, patients 1–5.

Reference

1. Weaver D, Novak-Frazer L, Palmer M, Richardson M, Bromley M, Bowyer P. Development of a novel mycobiome diagnostic for fungal infection. BMC Microbiol. 2024;24:63. PubMed https://doi.org/10.1186/s12866-024-03197-5
